# Postponed Application of Phosphorus and Potassium Fertilizers Mitigates the Damage of Late Spring Coldness by Improving Winter Wheat Root Physiology

**DOI:** 10.3390/plants13162311

**Published:** 2024-08-20

**Authors:** Hao Fang, Jinwei Huang, Xiatong Zhu, Muhammad Ahmad Hassan, Jin Ren, Jingyao Huang, Baoqiang Zheng, Xiang Chen, Feifei Lin, Jincai Li

**Affiliations:** 1 College of Agronomy, Anhui Agricultural University, Hefei 230036, China; fanghao@stu.ahau.edu.cn (H.F.); huangjinwei@stu.ahau.edu.cn (J.H.); zxt200074@stu.ahau.edu.cn (X.Z.); renjin0226@stu.ahau.edu.cn (J.R.); jingyaohuang@stu.ahau.edu.cn (J.H.); zhengbaoqiang@ahau.edu.cn (B.Z.); cx2468@ahau.edu.cn (X.C.); 2 Rice Research Institute, Anhui Academy of Agricultural Sciences, Hefei 230041, China; ahmaduaf93@stu.ahau.edu.cn; 3 Jiangsu Collaborative Innovation Centre for Modern Crop Production, Nanjing 210095, China

**Keywords:** winter wheat, late spring coldness, optimizing phosphorus and potassium application, root physiology, nutrient accumulation

## Abstract

Late spring coldness (LSC) is the main limiting factor threatening wheat yield and quality stability. Optimal nutrient management is beneficial in mitigating the harms of LSC by improving wheat root physiology. This study proposed a nutrient management strategy that postponed the application of phosphorus (P) and potassium (K), effectively strengthening wheat’s defense against LSC. This experiment used the winter cultivar “Yannong19” (YN 19) as plant material for two consecutive years (2021–2022 and 2022–2023). Two fertilizer treatments were used: traditional P and K fertilizers application (R1: base fertilizer: jointing fertilizer = 10:0) and postponed P and K fertilizers application (R2: base fertilizer: jointing fertilizer = 5:5); wheat plants at the anther connective formation stage shifted to temperature-controlled phytotrons for normal (T0, 11 °C/4 h) and low temperatures (T1, 4 °C/4 h; T2, −4 °C/4 h) as treatments of LSC. The results showed that under low temperature (LT) treatment, compared with R1, the R2 treatment increased the concentrations of osmotic adjustment substances (soluble sugars and soluble protein contents by 6.2–8.7% and 3.0–8.9%), enhanced activities of antioxidant enzymes (superoxide dismutase, peroxidase and catalase activities by 2.2–9.1%, 6.2–9.7% and 4.2–8.4%), balanced the hormone concentrations (increased IAA and GA_3_ contents by 2.8–17.5% and 10.4–14.1% and decreased ABA contents by 7.2–14.3%), and reduced the toxicity (malondialdehyde, hydrogen peroxide content and O_2_·^−^ production rate by 5.7–12.4%, 17.7–22.8% and 19.1–19.1%) of the cellular membranes. Furthermore, the wheat root physiology in R2 significantly improved as the root surface area and dry weight increased by 5.0–6.6% and 4.7–6.6%, and P and K accumulation increased by 7.4–11.3% and 12.2–15.4% compared to R1, respectively. Overall, the postponed application of P and K fertilizers enhanced the physiological function of the root system, maintained root morphology, and promoted the accumulation of wheat nutrients under the stress of LSC.

## 1. Introduction

Wheat (*Triticum aestivum* L.) is the world’s most widely cultivated food crop, consumed by more than 35% of the world’s population, and is the second-highest grain-producing crop in China [1]. Wheat production is directly related to economic stability and food security; however, with the intensification of global warming in recent years, temperature fluctuations in spring increase the risk of wheat plants suffering from late spring coldness (LSC) stress [2]. The LSC is an important meteorological factor that adversely affects plant growth and development and causes severe serious economic losses to wheat production in China, the United States, Australia, and Europe [3,4,5]. It often occurs at the jointing and booting stages during the critical growth periods for the rapid growth and differentiation of young wheat spikelets, which were highly sensitive to low-temperature (LT) stress [6]. When the sensitive growth stage encounters meteorological disasters, yield and grain quality seriously decline [7]. Previous research studies have predominantly concentrated on analyzing the above-ground plant parts, such as leaves, young spikes, and peduncles, with limited attention to exploring the below-ground plant part, i.e., roots.

Roots play a crucial role in the active uptake of nutrients and water while engaging in various symbiotic interactions with the soil [8]. Root systems’ spatial and temporal distribution alters below-ground ecological interactions, ultimately impacting plant performance, survival, and productivity [9]. However, the causes of the damage to the root growth under LT stress have still not been explored well. Short-term or continuous LT stress causes morphological, physiological, and biochemical changes that negatively affect the growth and development of plants, resulting in significant yield losses [10]. Buriro et al. [11] found that LT stress reduced root length, fresh stem and root weight, and root dry weight in wheat. Therefore, LT stress limits the growth of roots, which may be related to the physiological damage of roots. Posmyk et al. [12] investigated the changes in antioxidant enzyme activity in soybean roots when exposed to cold. When crops were exposed to LT stress, the activities of SOD, POD, CAT, and MDA contents increased in plant root cells [13]. Additionally, the absorption of water and nutrients and the hormonal balance in roots are severely disturbed [14]. Therefore, it is imperative to improve cultivation techniques to enhance the cold resistance of wheat roots.

Implementing timely preventive measures before LT events is challenging due to their random and unpredictable nature. In this scenario, nutrient management is crucial for enhancing tolerance to extreme temperature stress with P and K playing an important role in mitigating such stresses [15]. The application of P and K fertilizers enhances the adaptation of plants to the external environment by participating in signaling, energy dynamics, and enzyme catalysis, significantly improving the uptake and utilization of water and nutrients in wheat roots [16,17,18]. Optimizing P fertilizer application increased ascorbic acid, soluble protein, and sugar levels in wheat plants throughout the seedling, tillering, and jointing stages. This enhancement is crucial in improving wheat cold resistance and yield enhancement [19]. K alleviates freeze-induced cell dehydration and regulates the osmotic potential of plants under LT and freezing stress [20]. However, most existing reports on improving cold hardiness in wheat with P and K fertilizers concentrate mainly on the separate effects of each element or on the independent application of P and K fertilizers [21]. Considering that late spring cold primarily occurs during the jointing stage in the Huang Huai Hai wheat region, and conventional fertilization is also conducted at this stage, it is advantageous to concurrently administer nitrogen (N), P, and K fertilizers while ensuring consistent workload and fertilizer application quantities. This study proposed a nutrient management strategy that can effectively mitigate the detrimental impact of low temperatures on wheat. However, the specific mechanism by which postponing the application of P and K fertilizers alleviates the damage caused by LSC in wheat roots remains poorly comprehended.

In the present study, we hypothesized that the postponed application of P and K fertilizers would enhance wheat resilience to LT stress during the anther connective formation stage, improve root physiology, and increase P and K accumulation. Therefore, we performed a two-year pot experiment in which two P and K fertilizer application modes were opted under LSC at the anther connective formation stage. In conjunction with LSC and the treatment involving P and K fertilizers application, the study aimed to (1) investigate the impact of postponed application of P and K fertilizers on the root morphological traits, (2) analyze its effects on the roots physiology, i.e., activities antioxidant enzymes, osmotic adjustments, and hormonal balance; and (3) assess the advantageous outcomes of postponed application of P and K fertilizers on root nutrient accumulation during the anther connective formation stage. This study provided a theoretical basis and extended the technical support for optimizing P and K fertilizers to cope with LSC in the field conditions of China’s Huang-Huai-Hai wheat-growing region.

## 2. Results

### 2.1. Morphological Traits of Wheat Roots

**Figure 1:**
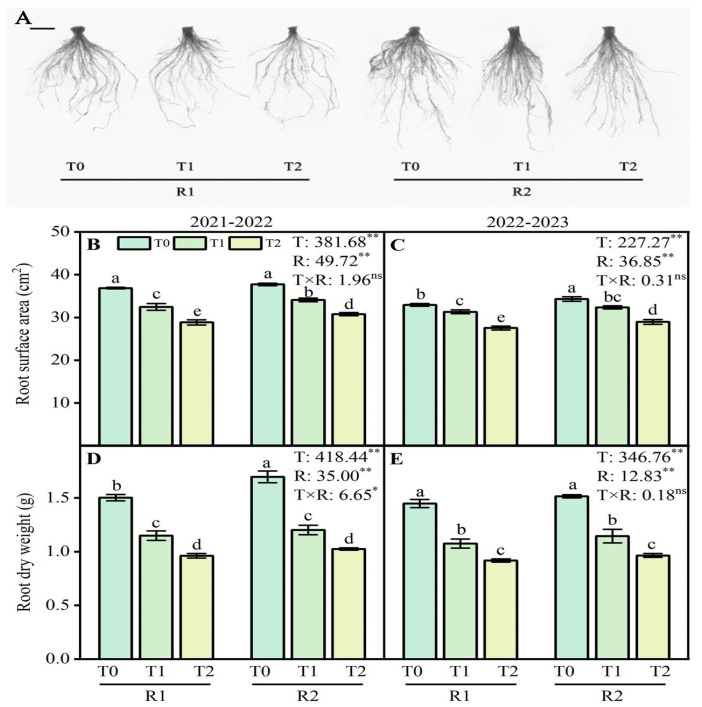
Effects of postponed application of P and K fertilizers on root morphology and dry weight under LT treatments. (**A**) Root morphology of different treatments; (**B**,**C**) difference in the response of root surface area to low temperature and top-dressing PK fertilizers mode at the anther connective formation stage during 2021–2022 and 2022–2023; (**D**,**E**) difference in the response of root dry weight to low temperature and top-dressing PK fertilizers at the anther connective formation stage during 2021–2022 and 2022–2023. T0, 11 °C; T1, 4 °C; T2, −4 °C; R1, traditional P and K fertilizers application; R2, postponed the application of P and K fertilizers; respectively. Different letters represent significant differences for comparing the six groups (LSD–test), *p*-values of multiple comparisons, *p* < 0.05. Analysis of variance with temperature (T) and PK fertilizers application (R) as two factors to analyze effects of single factor and interactions in randomized block experiments, ** *p* < 0.01, * *p* < 0.05, and ^ns^ *p* > 0.05.

The application methods of PK fertilizers and LT treatments affected the root surface area (RSA) and root dry weight (RDW) in varying intensities (Figure 1B–E). This study’s LT treatment significantly inhibited wheat root growth, resulting in low RSA and RDW (Figure 1A). Specifically, LT treatment reduced the RSA and RDW with a more significant decrease observed at lower temperatures. Compared with T0 treatment, T1 and T2 treatments reduced RSA by 11.9–21.7% and 4.9–16.4% during two growing seasons, respectively (Figure 1B,C). For RDW, the variation was similar to that for the RSA (Figure 1D,E). Compared with R1 treatment, the application of R2 at T1 and T2 enhanced RSA by 5.0–6.6% and RDW by 4.7–6.6%, respectively, during the growing season (2021–2022) (Figure 1B,D). Similar results were observed at the RSA and RDW in 2022–2023 (Figure 1C,E).

### 2.2. Soluble Sugar and Soluble Protein Content of Wheat Roots

**Figure 2:**
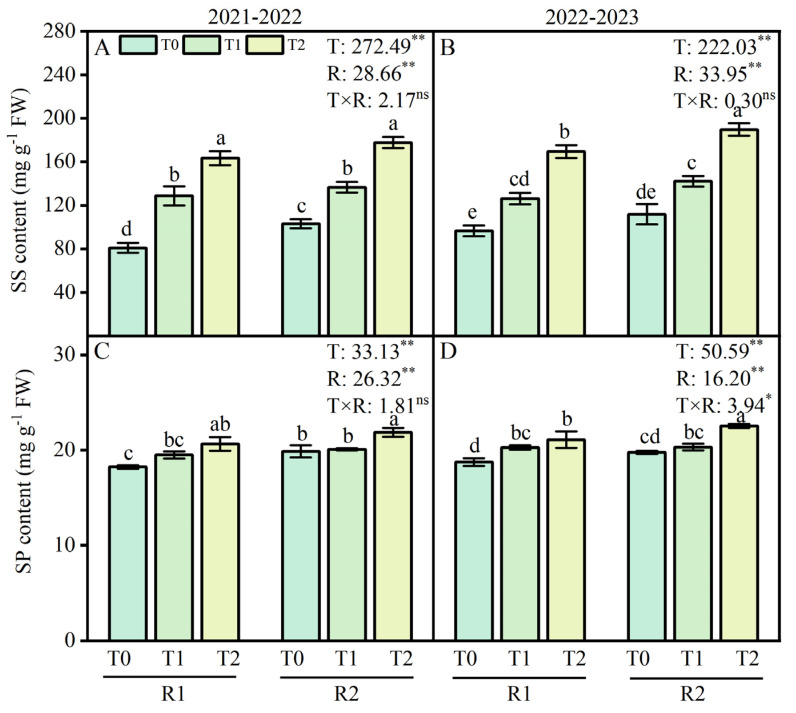
Effects of postponed application of P and K fertilizers on soluble sugar (SS) and soluble protein (SP) contents in wheat root under LT treatments. (**A**,**B**) SS content in root at anther connective formation stage during 2021–2022 and 2022–2023. (**C**,**D**) SP content in root at anther connective formation stage during 2021–2022 and 2022–2023. T0, 11 °C; T1, 4 °C; T2, −4 °C; R1, traditional P and K fertilizers application; R2, postponed the application of P and K fertilizers; respectively. Different letters represent significant differences for comparing the six groups (LSD–test), *p* values of multiple comparisons, *p* < 0.05. Analysis of variance with temperature (T) and PK fertilizers application (R) as two factors to analyze the effects of single factor and interactions in randomized block experiments, ** *p* < 0.01, * *p* < 0.05, and ^ns^ *p* > 0.05.

Wheat roots soluble sugar (SS) content increased, but soluble protein (SP) content decreased following LT treatment at the anther connective formation stage (Figure 2). Compared with the T0 treatment, SS contents increased as the LT decreased; however, the opposite trend was observed for SP contents. Our study showed that compared with the T0 treatment, the LT treatment at T1 and T2 increased SS contents by 58.9–101.7% and 30.7–75.4% during two growing seasons, respectively. Additionally, the contents of SP decreased by 5.5–11.7% and 3.9–11.1% at T1 and T2 under LT treatments in 2021–2022 and 2022–2023, respectively. Compared with the R1 treatment, applying R2 at T1 and T2 enhanced SS contents by 6.2–8.7% and SP contents by 3.0–8.9% during the 2021–2022 growing season (Figure 2A,C). Similar results were observed at the SS and contents in roots during 2022–2023 (Figure 2B,D).

### 2.3. Antioxidant System Enzyme Activities of Wheat Roots

**Figure 3:**
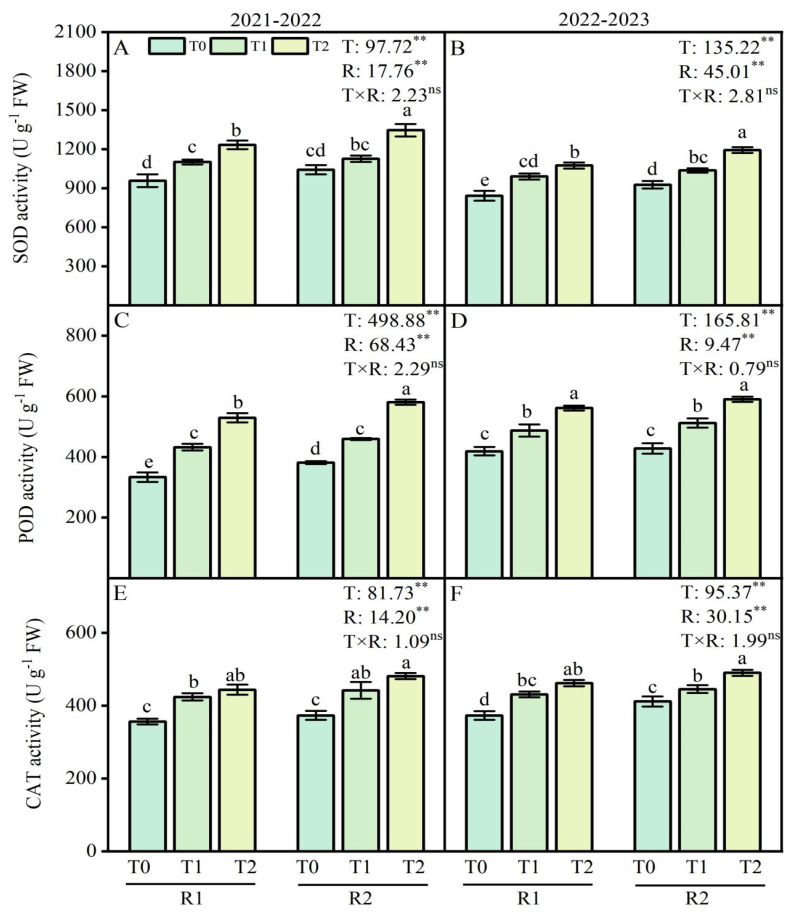
Effects of postponed application of P and K fertilizers on superoxide dismutase (SOD), peroxidase (POD), and catalase (CAT) activities in wheat root under LT treatments. (**A**,**B**) The activity of SOD in root at anther connective formation stage during 2021–2022 and 2022–2023. (**C**,**D**) The activity of POD in root at anther connective formation stage during 2021–2022 and 2022–2023. (**E**,**F**) The activity of CAT in root at anther connective formation stage during 2021–2022 and 2022–2023. T0, 11 °C; T1, 4 °C; T2, −4 °C; R1, traditional P and K fertilizers application; R2, postponed application of P and K fertilizers; respectively. Different letters represent significant differences for comparing the six groups (LSD–test), *p* values of multiple comparisons, *p* < 0.05. Analysis of variance with temperature (T) and PK fertilizers application (R) as two factors to analyze the effects of single factor and interactions in randomized block experiments, ** *p* < 0.01, and ^ns^ *p* > 0.05.

Analysis of variance (ANOVA) revealed that the LSC temperature and PK fertilizers application treatments had a significant effect on the superoxide dismutase (SOD), peroxidase (POD), and catalase (CAT) activity, while their interaction had no significant impact on the activities of SOD, POD, and CAT (Figure 3). In this study, compared with the T0 treatment, the LT treatments at T1 and T2 increased SOD activity by 15.0–28.7% and 17.6–27.6% in 2021–2022 and 2022–2023, respectively. Additionally, the activity of POD increased by 29.7–58.8% and 16.3–33.9% at T1 and T2 under LT treatment in 2021–2022 and 2022–2023 (Figure 3A–D). For the CAT activity of wheat root, the variation was similar to that for the activities of SOD and POD (Figure 3E,F). Compared with R1 treatment, R2 treatment at T1 and T2 enhanced SOD activity by 2.2–9.1%, POD activity by 6.2–9.7%, and CAT activity by 4.2–8.4%, respectively, during 2021–2022 (Figure 3A,C,E). Similar results were observed at the SOD, POD, and CAT activity in 2022–2023 (Figure 3B,D,F).

### 2.4. ROS Contents of Wheat Roots

**Figure 4:**
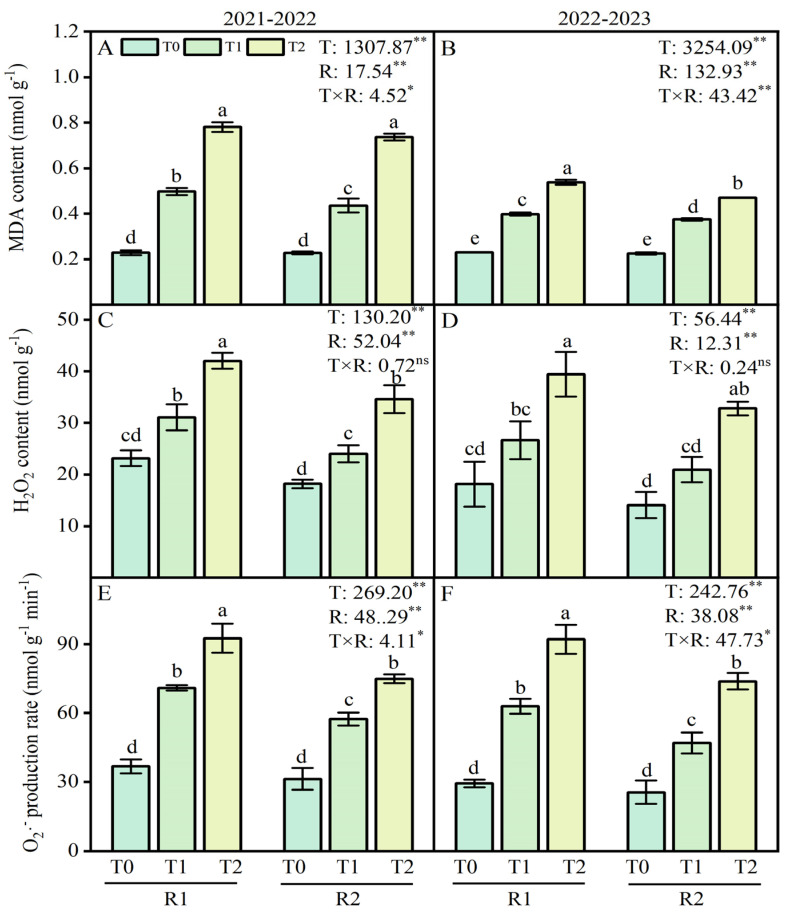
Effects of postponed application of P and K fertilizers on MDA, H_2_O_2_ content and O_2_·^−^ production rate in wheat root under LT treatments. (**A**,**B**) MDA content in root at anther connective formation stage during 2021–2022 and 2022–2023. (**C**,**D**) H_2_O_2_ content in root at anther connective formation stage during 2021–2022 and 2022–2023. (**E**,**F**) O_2_·^−^ production rate in root at anther connective formation stage during 2021–2022 and 2022–2023. T0, 11 °C; T1, 4 °C; T2, −4 °C; R1, traditional P and K fertilizers application; R2, postponed the application of P and K fertilizers; respectively. Different letters represent significant differences for comparing the six groups (LSD–test), *p* values of multiple comparisons, *p* < 0.05. Analysis of variance with temperature (T) and PK fertilizers application (R) as two factors to analyze effects of single factor and interactions in randomized block experiments, ** *p* < 0.01, * *p* < 0.05, and ^ns^ *p* > 0.05.

ANOVA revealed that the LSC temperature and PK fertilizer application treatments had a significant effect on the malondialdehyde (MDA) content, hydrogen peroxide (H_2_O_2)_ content, and O_2_·^−^ production rate, while their interaction had a significant impact on the MDA content and O_2_·^−^ production rate (Figure 4). In this study, compared with the T0 treatment, the LT treatments at T1 and T2 increased MDA contents by 117.9–242.1% and 73.1–133.7% in 2021–2022 and 2022–2023, respectively. Additionally, the content of H_2_O_2_ increased by 34.2–81.5% and 46.9–117.5% at T1 and T2 under LT treatments in 2021–2022 and 2022–2023 (Figure 4A–D). The variation for wheat root’s O_2_·^−^ production rate was similar to the content of MDA and H_2_O_2_ (Figure 4E,F). Compared with the R1 treatment, the application of R2 at T1 and T2 decreased the MDA contents by 5.7–12.4%, the contents of H_2_O_2_ by 17.7–22.8%, and the O_2_·^−^ production rate by 19.1% during the 2021–2022 growing season (Figure 4A,C,E). Similar results were observed for the MDA content, H_2_O_2_ contents, and O_2_·^−^ production rate in 2022–2023 (Figure 4B,D,F).

### 2.5. Endogenous Hormone Content of Wheat Roots

**Figure 5:**
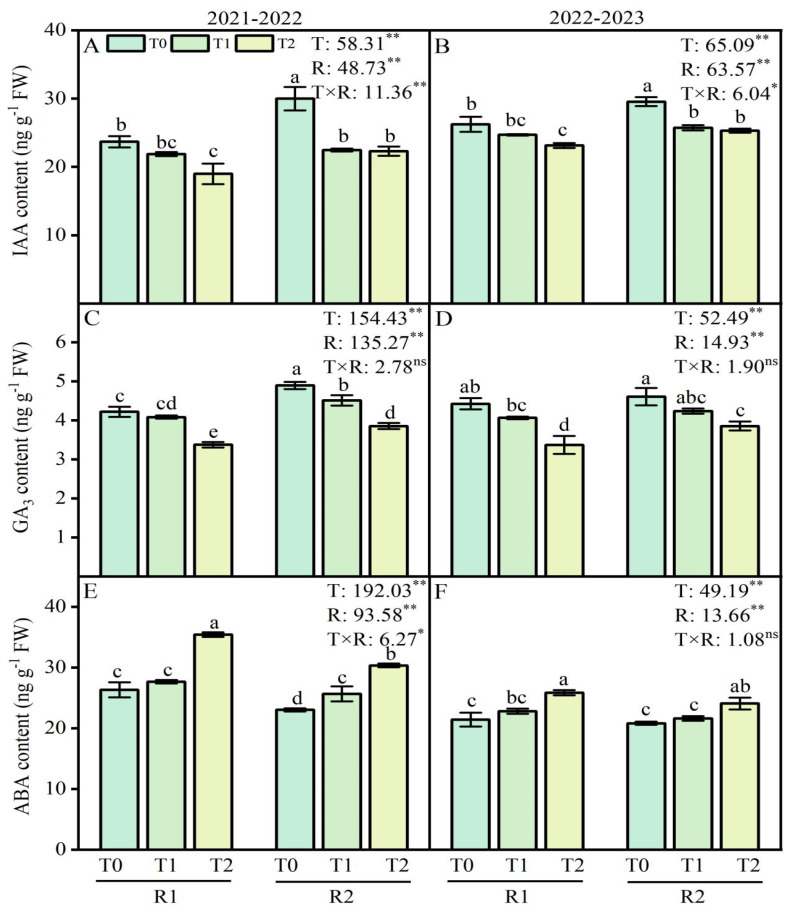
Effects of postponed application of P and K fertilizers on IAA, GA_3,_ and ABA content in wheat root under LT treatments. (**A**,**B**) IAA content in root at anther connective formation stage during 2021–2022 and 2022–2023. (**C**,**D**) GA_3_ content in root at anther connective formation stage during 2021–2022 and 2022–2023. (**E**,**F**) ABA content in root at anther connective formation stage during 2021–2022 and 2022–2023. T0, 11 °C; T1, 4 °C; T2, −4 °C; R1, traditional P and K fertilizers application; R2, postponed application of P and K fertilizers; respectively. Different letters represent significant differences for comparing the six groups (LSD–test), *p* values of multiple comparisons, *p* < 0.05. Analysis of variance with temperature (T) and PK fertilizers application (R) as two factors to analyze effects of single factor and interactions in randomized block experiments, ** *p* < 0.01, * *p* < 0.05, and ^ns^ *p* > 0.05.

The ANOVA results revealed that the LSC temperature and PK fertilizer application treatments had a significant effect on the IAA, GA_3,_ and ABA contents, while their interaction had a significant impact on the IAA content (Figure 5). The ABA content increased in wheat roots, while the IAA and GA_3_ content decreased following LT treatments at the anther connective formation stage. The contents of IAA and GA_3_ showed a decreasing trend as the LT decreased. However, the opposite trend was observed for ABA contents. In this study, compared with the T0 treatment, the LT treatment at T1 and T2 decreased the IAA content by 7.7–19.9% and 5.8–11.8% during 2021–2022 and 2022–2023, respectively. Additionally, the contents of GA_3_ increased by 3.3–20.0% and 8.2–23.8% at T1 and T2 under LT treatment in 2021–2022 and 2022–2023 (Figure 5A–D). However, the opposite trend was observed for ABA contents (Figure 5E,F). Compared with the R1 treatment, the application of R2 at T1 and T2 increased the IAA content by 2.8–17.5% and the content of GA_3_ by 10.4–14.1%, respectively, during the 2021–2022 growing season (Figure 5A,C). However, compared with the R1 treatment, the application of R2 at T1 and T2 decreased the ABA content by 7.2–14.3%. Similar results were observed for the IAA, GA_3,_ and ABA contents in 2022–2023 (Figure 5B,D,F).

### 2.6. Acid Phosphatase and Alkaline Phosphatase Activities of Wheat Roots

**Figure 6:**
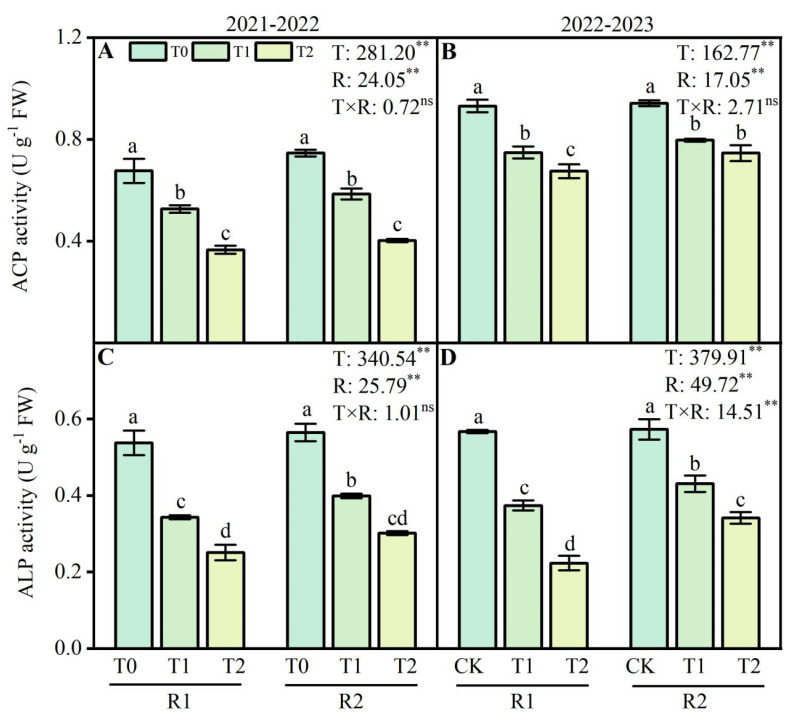
Effects of postponed application of P and K fertilizers on acid phosphatase (ACP) and alkali phosphatase (ALP) activity in wheat root under LT treatments. (**A**,**B**) The activity of ACP in root at anther connective formation stage during 2021–2022 and 2022–2023. (**C**,**D**) The activity of ALP in root at anther connective formation stage during 2021–2022 and 2022–2023. T0, 11 °C; T1, 4 °C; T2, −4 °C; R1, traditional P and K fertilizers application; R2, postponed application of P and K fertilizers; respectively. Different letters represent significant differences for comparing the six groups (LSD –test), *p* values of multiple comparisons, *p* < 0.05. Analysis of variance with temperature (T) and PK fertilizers application (R) as two factors to analyze effects of single factor and interactions in randomized block experiments, ** *p* < 0.01, and ^ns^ *p* > 0.05.

Wheat root acid phosphatase (ACP) and alkaline phosphatase (ALP) activity decreased following LT treatments at the anther connective formation stage (Figure 6). This study showed that compared with the T0 treatment, T1 and T2 treatments decreased ACP activity by 22.2–45.9% and 19.7–27.5%, respectively, during 2021–2022 and 2022–2023. Additionally, the activity of ALP decreased by 36.1–53.3% and 34.1–60.7% at T1 and T2 under LT treatments in 2021–2022 and 2022–2023. Compared with the R1 treatment, the application of R2 at T1 (4 °C) and T2 (−4 °C) enhanced the activity of ACP by 10.1–11.2% and ALP activity by 16.2–20.2% during the 2021–2022 growing season (Figure 6A,C). Similar results were observed for the ACP and ALP activity in 2022–2023 (Figure 6B,D).

### 2.7. Phosphorus Accumulation and Potassium Accumulation Contents of Wheat Roots

**Figure 7:**
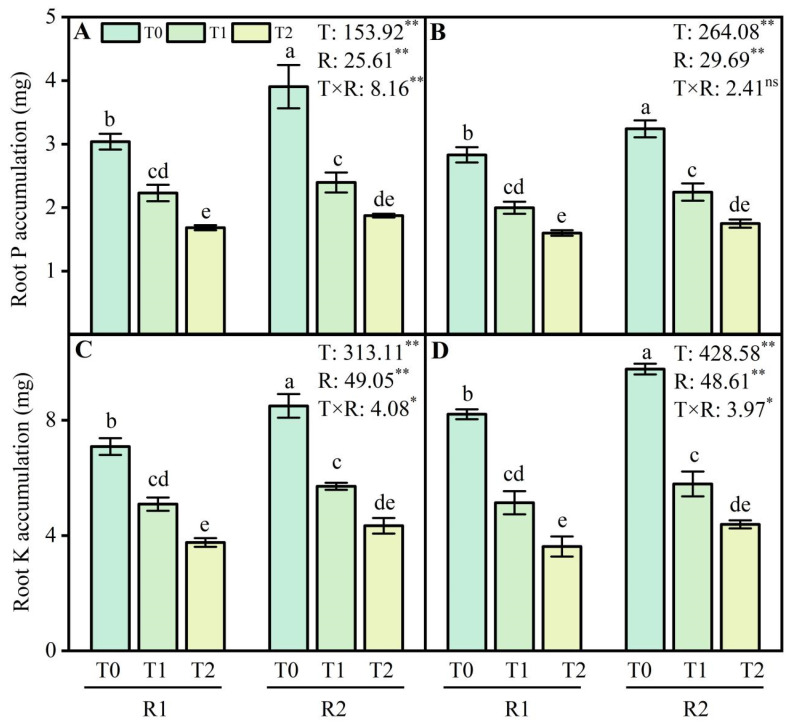
Effects of postponed application of P and K fertilizers on P and K accumulation in wheat root under LT treatments. (**A**,**B**) The content of root P accumulation at anther connective formation stage during 2021–2022 and 2022–2023. (**C**,**D**) The content of root K accumulation at anther connective formation stage during 2021–2022 and 2022–2023. T0, 11 °C; T1, 4 °C; T2, −4 °C; R1, traditional P and K fertilizers application; R2, postponed application of P and K fertilizers; respectively. Different letters represent significant differences for comparing the six groups (LSD-test), *p* values of multiple comparisons, *p* < 0.05. Analysis of variance with temperature (T) and PK fertilizers application (R) as two factors to analyze effects of single factor and interactions in randomized block experiments, ** *p* < 0.01, * *p* < 0.05, and ^ns^ *p* > 0.05.

Wheat roots’ phosphorus accumulation (PA) and potassium accumulation (KA) contents decreased following LT treatments at the anther connective formation stage (Figure 7). This study showed that compared with the T0 treatment, T1 and T2 treatments decreased the PA content by 26.6–44.6% and 29.4–43.4% during the two growing seasons, respectively (Figure 7A,B). Additionally, the content of KA decreased by 28.2–47.0% and 37.4–55.9% at T1 and T2 under LT treatment in 2021–2022 and 2022–2023 (Figure 7C,D). Compared with the R1 treatment, applying R2 at T1 and T2 enhanced the PA content by 7.4–11.3% and KA content by 12.2–15.4% during the 2021–2022 growing season (Figure 7A,C). Similar results were observed in PA and KA in 2022–2023 (Figure 7B,D).

### 2.8. Correlation Coefficients between Root Morphological Traits, Antioxidant Enzymes, Osmotic Adjustment Substance, Endogenous Hormone, Phosphatase Activity, and Nutrient Accumulation

**Figure 8:**
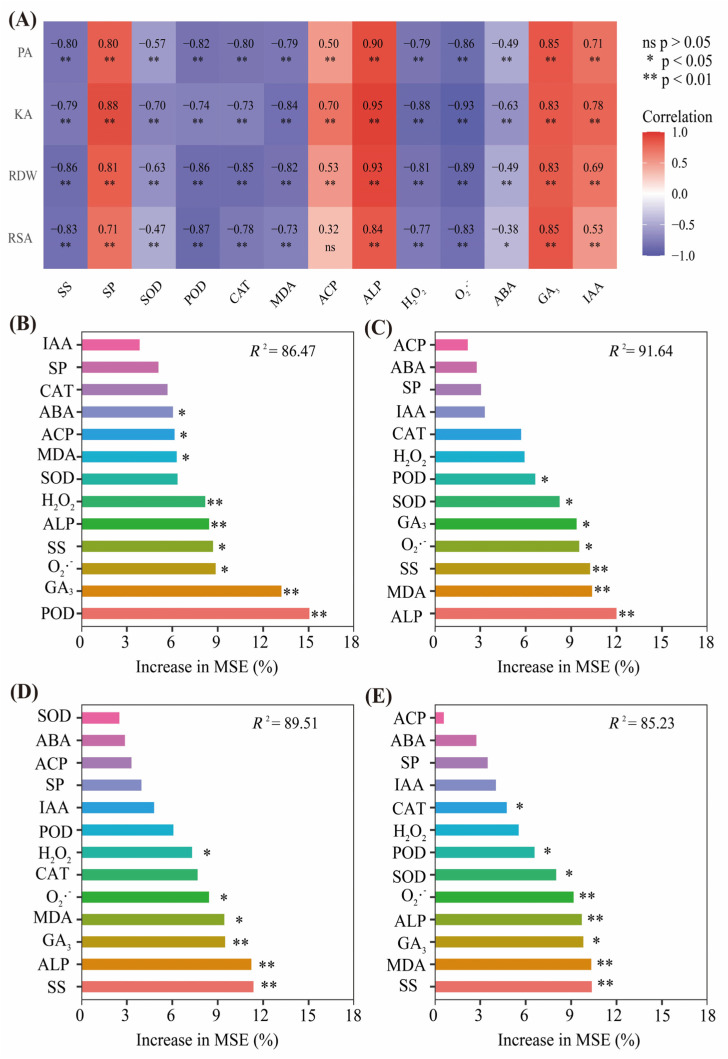
(**A**) The heatmap of correlations among PA, KA, RSA, RDW, and physiological parameters of roots under different treatments. The relative importance (%) of variables for the physiological parameter response to (**B**) RSA, (**C**) RDW, (**D**) KA, and (**E**) PA based on the random forest regression model. PA: phosphorus accumulation; KA: potassium accumulation; RDW, root dry weight; RSA: root surface area; SS: soluble sugar; SP: soluble protein; SOD, superoxide dismutase; POD, peroxidase; CAT, catalase; MDA, malondialdehyde; ACP, acid phosphatase activity; ALP, alkali phosphatase activity; H_2_O_2,_ hydrogen peroxide; O_2_^·−^, rate of superoxide anion generation; ABA, abscisic acid; GA_3_, gibberellic acid; IAA, auxin under different treatments. ns indicates a non-significant correlation. * and ** represent significant correlations at 5% and 1% probability levels, respectively.

According to Figure 8, the RSA and RDW are positively correlated with the contents of SP, GA3, IAA, and the activities of ACP and ALP. The P and K accumulation are also positively correlated with the contents of SP, GA_3_, IAA, and the activities of ACP and ALP. The P accumulation is positively correlated with ACP and ALP activities, which indicates that P metabolizing enzyme activity promotes P accumulation in the root system. However, the P and K accumulation exhibited significant negative correlations with the SOD, POD, CAT, MDA, and H_2_O_2_ contents as well as the O_2_·^−^ production rate. These results indicated that P and K accumulations were primarily influenced by cell membrane and antioxidant activities. However, the IAA and GA_3_ contents were positively correlated with root P and K accumulation. The opposite trend was observed for ABA contents. Those results indicated that excessive ABA accumulation suppresses endogenous hormone balance, and the balance between endogenous hormones affects P and K accumulation in the root.

The random forest (RF) analysis demonstrated that POD and GA_3_ were the crucial drivers of the variations in RSA (Figure 8B), whereas the RDW was mainly governed by ALP and MDA (Figure 8C). The RF analysis demonstrated that SS and ALP were the crucial drivers of the variations in KA (Figure 8D), whereas the RDW was mainly governed by SS and MDA (Figure 8E). 

## 3. Discussion

### 3.1. Optimizing P and K Application Improved the Growth of Wheat Root Under LT Stress 

LT stress affected wheat plants’ growth and development with roots typically subjected to more abiotic stress than shoots [11,22]. Hence, it is critical to improve the resistance of wheat roots to LT stress, which is of great significance for disaster prevention and mitigation in wheat production. This study indicated that LT stress decreased the accumulation of wheat root biomass during the anther connective formation stage (Figure 1D,E), which is consistent with previous studies [23]. The decreased wheat root activity slowed root growth and reduced biomass accumulation [10]. The root system is the main organ of wheat that absorbs water and mineral nutrients in the soil, and its growth status will affect the growth and development of the wheat shoot system [24]. This study significantly reduced the root surface area under LT stress (Figure 1B,C). A similar result was reported in rice; cold-sensitive genotypes display a reduced root dry weight and shorter and fewer root hairs associated with a smaller root area [25]. Moreover, chilling stress can decrease the root hydraulic conductance, water uptake, water content, and nutrient uptake [26]. The root system architecture, such as higher root density and more lateral root branches, enables roots to take up more P from the topsoil layer [27,28]. Applying P fertilizer boosts below-ground microbial activity, fostering mutually beneficial conditions for robust root growth and development [29]. Increased microbial activity excites soil temperature to a certain extent and alleviates the adverse impacts of LT stress [21]. Applying P fertilizer increases the root surface area, supporting active nutrient–water uptake [30]. K is engaged in nearly all the plant’s physiological processes requiring water [31]. Nutrient management is one of the best options in response to extreme temperature stress tolerance, and among all the nutrients, K plays a significant role in coping with temperature stress [20]. K helps to activate the various physiological and metabolic processes [32]. Several field trials in various crops have also shown a similar phenomenon in which a sufficient K supply can eliminate frost damage [33]. These results indicated that the postponed application of P and K fertilizers alleviated the root morphology of wheat plants after LSC damage, including root surface area and dry weight. So, the postponed application of P and K fertilizers was conducive to maintaining the balance between root and shoot growth by boosting nutrient accumulation and promoting wheat development.

### 3.2. Optimizing P and K Application Alleviated Root Physiology in Wheat under LT Stress 

The MDA content, H_2_O_2_ concentrations, and O_2_·^−^ production rate in wheat root were decreased under LT stress by the postponed P and K fertilizers application in the present experiment (Figure 4A–F). Optimizing P and K applications have lower levels of MDA [34]. In contrast, producing reactive oxygen species (ROS) during cold leads to damage in roots, as illustrated by higher lipid peroxidation levels and H_2_O_2_ accumulation in cold-sensitive rice genotypes [35].

Under LT treatments, the antioxidant system of wheat roots was activated, and the antioxidant enzyme activity increased [36]. The induction of antioxidant enzymes in response to cold stress by mineral nutrients facilitates eliminating ROS and enhancing the antioxidant defense against oxidative stress in the root [37]. In this study, antioxidant enzyme activities (SOD, POD, CAT) were significantly increased in wheat roots under LT stress by the postponed application of P and K fertilizer with variations among the different temperature treatments (Figure 3A–F). The previous study found that compared with traditional phosphorus application, the activities of SOD, POD, and CAT of twice-split phosphorus application were significantly increased on the day of LT treatment, respectively [34,38].

Plants have developed various defense systems to respond to cold stress [39]. The regulation of signaling by plant hormones is crucial for antioxidant defense against stressors [40]. In plants, such as ABA, GA, and IAA, endogenous hormones can act as signaling molecules to participate in cold resistance [41]. The results showed that under LT stress, postponed P and K fertilizer application increased the IAA and GA_3_ contents in wheat roots while ABA content decreased (Figure 5A–F). LT-induced ABA accumulation in winter wheat at the booting stage altered the activity of enzymes related to sucrose metabolism, which led to sucrose synthesis and accumulation in the young ears, thus causing yield losses [42]. In this study, the IAA and GA_3_ concentrations inversely altered to the concentrations of ABA not only after LT but also after the postponed application of P and K fertilizers. In addition, the postponed application of P and K fertilizers maintained lower levels of endogenous ABA and higher GA_3_ and IAA levels, which changed the balance of these hormones to adapt to LT stress (Figure 5A–F).

The findings suggest that the postponed application of P and K fertilizers enhanced the cold resistance of wheat roots through a combination of physiological effects, such as antioxidant capacity, membrane lipid protection, and regulation of plant hormones. The equilibrium among these factors reinforces cold resilience.

### 3.3. Optimizing P and K Application Enhanced Phosphatase Activity and Nutrient Accumulation in Wheat Root under LT Stress

The plant root system is vital for efficient soil water, nutrient uptake, and stress sensing [43]. However, the intake of water and nutrients from the root system is limited in LT environments. LSC is harmful to the growth and development of wheat and the accumulation of nutrients, mainly affecting the metabolism of the root system and nutrient absorption and utilization [17]. Wheat root activity, ACP, and ALP are important in mineral nutrient and water uptake and conductance [44]. The metabolic activities of ACP and ALP in wheat roots were inhibited under the harm of LSC, which led to the decrease in the P absorption capacity of wheat roots and affected the metabolic process of P [34]. In this study, the ACP and ALP activity in wheat root were significantly decreased under LT stress by the postponed application of P and K fertilizers (Figure 6A–D). Under a low root zone temperature, the ability of roots to take up mineral nutrients is significantly decreased because of the reduced activity of enzymes and transporters [25]. LT stress may reduce the absorption efficiency of P in wheat roots, thus reducing the accumulation of P in wheat roots. However, mineral nutrients positively affect the amelioration of cold stress in plants [45]. Optimizing P application increased root ACP activity, promoted nutrient accumulation and transport, and alleviated late spring coldness’s harm [34]. The ACP activity in the root system contributes to the activation of P and the symbiotic relationship between beneficial microorganisms [46]. Increasing P activity is plants’ primary physiological mechanism of P mobilization [47]. Applying P and K to wheat is an effective nutrient management strategy for nitrogen use efficiency improvement, N losses, and soil N accumulation in a rice–wheat system [48]. Xu et al. [34] pointed out that twice-split phosphorus application increased phosphorus accumulation, and the translocation and partitioning to grains also enhanced after flowering, with the phosphorus harvest index increasing by 2.7–3.1% in the twice-split P application treatments compared with that in the conventional phosphorus application treatments at the low temperature. Various field experiments on different crops have shown that sufficient K supply increases K accumulation and prevents freezing injury [49]. In this study, compared with the R1 treatment, the application of R2 at LT stress enhanced P accumulation by 7.4–11.3% and K accumulation by 12.2–15.4% during the wheat-growing season (Figure 7A–D). These findings suggest that the postponed application of P and K fertilizers protects the phosphatase activity of wheat roots, including ACP and ALP, enhancing the resistance of roots under LT stress and ensuring nutrient accumulation.

## 4. Materials and Methods

### 4.1. Plant Growth Conditions

**Figure 9:**
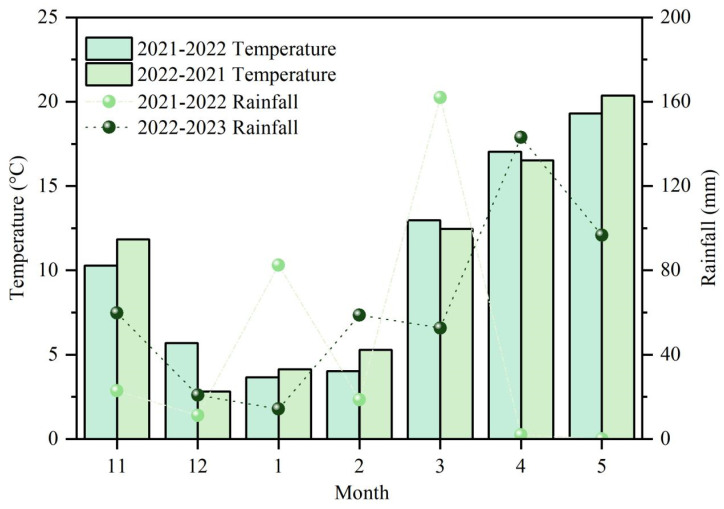
Temperature and precipitation during the wheat growth seasons.

Pot experiments were conducted in the Nongcui Garden of the Anhui Agricultural University (31°8′ N, 117°2′ E) in 2021–2022 and 2022–2023 in Hefei, Anhui Province, China. The subtropical humid monsoon climate zone was selected as the test site. The meteorological data of the wheat growing seasons in this study, including the mean daily temperature and the mean monthly rainfall, are summarized in Figure 9. The potting soil type is yellow–brown and taken from a 0–20 cm cultivation layer. The nutrient contents of the experimental field before sowing are shown in Table 1.

### 4.2. Experimental Design and Cropping Management

The high-yield wheat cultivar Yannong 19, which is widely planted in the Huang-Huai-Hai wheat growing area of China, was used in this study. Wheat seeds were sown on 14 November 2021 and 11 November 2022, and harvest time for the two years was 21 May and 18 May. A two-factor experiment with a completely randomized block was designed, including temperature and PK fertilizers application treatment. The pots were 35 cm high and 26 cm in diameter. Each pot was filled with 10 kg of sieved soil, and then 3 cm of soil was covered with wheat seeds after sowing. 

Traditionally, field fertilization was applied, and the P and K fertilizers were used as base fertilizers before sowing. Nitrogen was applied to the soil twice: (i) before sowing and (ii) at the jointing stage. The fertilizers used in the experiment were urea (N 46%), superphosphate (P_2_O_5_ 12%), and potassium chloride (K_2_O 60%). In this experiment, 1.8 g urea (base application of 1.2 g before sowing + top-dressing application of 0.6 g at the jointing stage) was applied to each pot during the growth period. Before sowing, the traditional P and K fertilizers treatment (R1) was used for all 5.0 g P and K fertilizers applications. The postponed P and K fertilizers treatment (R2) was applied with 2.5 g P and K fertilizers before sowing separately and at the jointing stage. The top dressing during the jointing stage was on March 11 and 15 in 2022 and 2023, respectively.

**Figure 10:**
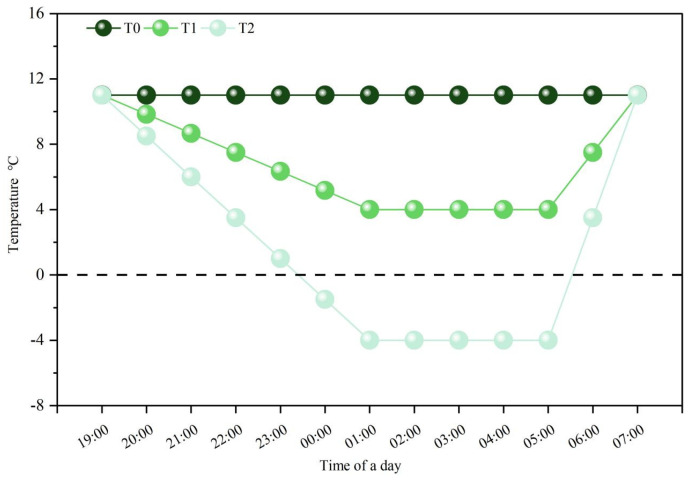
Dynamics of ambient temperature and in the artificial climate chamber during the experiment. T0, 11 °C; T1, 4 °C; T2, −4 °C.

We observed wheat plants’ different growth stages and patterns under a microscope (OLYMPUS SZ2-ILST; Tokyo, Japan). Moreover, all the pots, except control treatments, were shifted to an artificial climate chamber at the anther connective formation stage. To better understand the natural conditions of LT variations, the artificial climate chamber temperature gradually declined from ambient temperature 11 °C (T0) to target LT 4 °C (T1), −4 °C (T2) in 6 h duration, was subjected to LT treatments for the next 4 h, and then increased to the control temperature (Figure 2). Humidity in the chamber was kept at 75%, and light intensity was maintained at 0 µmol m^−2^ s^−1^ over both years (Figure 10). The wheat plants were then kept in the field until maturity. The experiment comprised 6 treatments [(2 fertilization treatments: R1, R2) × (3 temperature treatments: T0, T1, T2) = 6]. Each treatment contained 10 pots and was replicated three times (30 pots for each treatment); there were 180 pots in total. Eighteen seeds were sown in each pot, and nine wheat plants were maintained at the three-leaf stage through thinning. 

### 4.3. Sampling and Measurements

After LT treatment, three plants were randomly sampled from each pot at the anther connective formation stage. Then, these samples proceeded to the necessary morphological and physiological measurements.

#### 4.3.1. Root Morphological Traits

The roots of sampled wheat plants were washed carefully for scanning using the photo scanner. The root morphological indices were determined by WinRhizo Pro software (v2009, Regent Instrument Inc, Quebec, QC, Canada), according to Armengaud et al. [50]. The scanned roots were placed in an oven at 75 °C until constant weight to determine the dry weight of the roots.

#### 4.3.2. Root Physiology

The wheat root’s physiological properties were evaluated under LT treatment at the anther connective formation stage. The wheat root samples were frozen in liquid nitrogen and then sealed in an ultra-low temperature refrigerator with tin foil to protect them from light. The SS and SP content and the activities of SOD, CAT, and POD of wheat root samples were measured according to the assay of Xu et al. [38]. MDA content was determined using the colorimetric thiobarbituric acid method, and the results were expressed as nmol g^−1^ (FW) [51]. H_2_O_2_ content was determined according to the method of Kamran et al. [52], and the results were expressed as μg g^−1^ (FW). The rate of superoxide anion (O_2_·^−^) production was determined by the hydroxylamine oxidation method [53], and the results were shown as mmol g^−1^ min^−1^ (FW). We analyzed the root activity of ACP and ALP using a plate reader (Multiskan FC, Thermo Fisher Scientific Company, Massachusetts, USA) with kits (Solarbio Science &Technology Company, Beijing, China). Phytohormone concentrations, including ABA, IAA, and GA_3_, were quantified using the methodology detailed by Li et al. [54].

#### 4.3.3. Root Nutrient Accumulation

To measure the root’s nutrients (P, K) accumulation, wheat root samples were dried and pulverized by a ball mill (MM400, Retsch Company, Arzberg, Germany) and then digested using the H_2_SO_4_-H_2_O_2_ method. A fully automated continuous flow analyzer determined the phosphorus content in the digest (AA3, Seal Company, Norderstedt, Germany), and the content of potassium was determined using a flame photometer (FP640, Precision Instrument Science Company, Shanghai, China) [34]. Moreover, the following parameters related to phosphorus and potassium accumulation within the wheat plants during grain filling were calculated:

Wheat root phosphorus (potassium) accumulation = Root dry matter weight × phosphorus (potassium) content

### 4.4. Statistical Analysis

Data were subjected to two-way analyses of variance (ANOVA) using the general linear model to calculate the effects on the measured parameters. Samples were analyzed in triplicate, and mean values were used to compare them. The analysis of variance was performed using the LSD (least significant difference) test in SPSS 26.0 (Statistical Product and Service Solutions, IBM, Armonk, NY, USA). Graphs were drawn using Origin 2021 (OriginLab Corporation, Northampton, MA, USA) and Adobe Photoshop 2021 (Adobe Systems Inc., San Jose, CA, USA).

## 5. Conclusions

**Figure 11:**
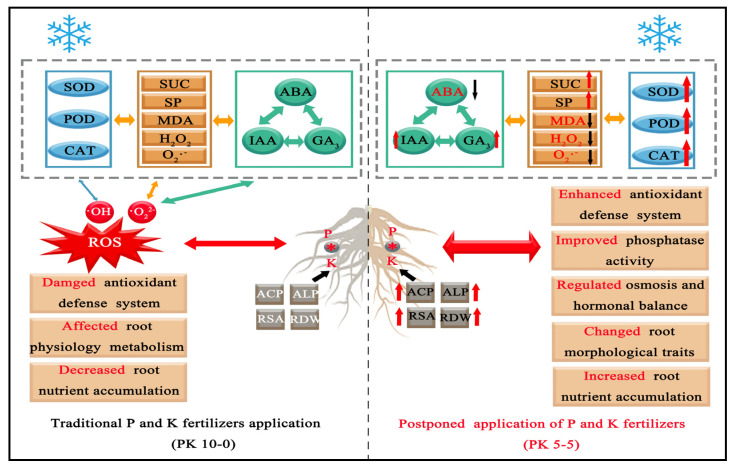
Schematic model of the mechanisms underlying the influence of root characteristics under LT stress by different P and K fertilizers treatment, antioxidant enzymes, osmotic adjustment substance, plant hormone, phosphatase activity, morphological traits, and nutrient accumulation. For LT treatment by postponed application of P and K fertilizers in the wheat root, the black arrows represent downregulation, and the red arrows represent upregulation.

Under LT treatment, wheat roots’ antioxidant capacity, active oxygen metabolism, and plant hormones were negatively affected, inhibiting root growth and nutrient accumulation. Our study confirmed that the postponed application of P and K fertilizers enhanced the antioxidant enzyme activity (SOD, POD, and CAT) and reduced membrane damage (MDA, H_2_O_2_ content, and O_2_·^−^ production rate). It improved the balance of wheat root’s ABA, IAA, and GA_3_. It also increased ACP and ALP activity, enhanced P and K accumulation, and improved the root surface and dry weight (Figure 11). These findings provide effective and practical knowledge and approaches to alleviate the effects of LSC on wheat production. Therefore, this study provides a theoretical basis for stress-resistant cultivation and the efficient utilization of fertilizers.

## Figures and Tables

**Table 1:** The nutrient content of the experimental field before sowing.

Year	Organ Matter (g·kg^−1^)	Total N (g·kg^−1^)	Available N (mg·kg^−1^)	Available P (mg·kg^−1^)	Available K (mg·kg^−1^)
2021–2022	16.5	1.3	112.2	23.0	161.6
2022–2023	16.1	1.0	110.4	26.2	149.5

## Data Availability

The data presented in this study are available upon request from the authors.
